# Accurate imputation of African cattle genomes using a diverse reference panel

**DOI:** 10.1186/s12864-026-12818-4

**Published:** 2026-05-06

**Authors:** Said I. Ng’ang’a, James A. Ward, Stephen J. Rossiter, Chris G. Faulkes, Katia Bougiouri, Gillian P. McHugo, Fenton P. D. Cotterill, Atunga Nyachieo, Olaf Thalmann, Ivica Medugorac, Stefan Krebs, Tad S. Sonstegard, Olivier Hanotte, Daniel G. Bradley, Gary Vaughan-Smith, David E. MacHugh, Laurent A.F. Frantz

**Affiliations:** 1 Palaeogenomics Group, Department of Veterinary Sciences, Ludwig Maximilian University, Munich, Germany; 2 School of Biological and Behavioural Sciences, Queen Mary University of London, London, UK; 3 Bioinformatics and Health Informatics Group, Institute of Primate Research, Nairobi, Kenya; 4 Animal Genomics Laboratory, UCD School of Agriculture and Food Science, University College Dublin, Dublin, Ireland; 5 Population Genomics Group, Department of Veterinary Sciences, Faculty of Veterinary Medicine, Ludwig Maximilian University of Munich, Munich, Germany; 6 Section for Molecular Ecology and Evolution, Globe Institute, University of Copenhagen, Copenhagen, Denmark; 7 SilverStreet Capital, London, UK; 8 Laboratory for Functional Genome Analysis, Gene Center, Ludwig Maximilian University of Munich, Munich, Germany; 9 Acceligen, Eagan, MN USA; 10 International Livestock Research Institute, Addis Ababa, Addis Ababa, Ethiopia; 11 School of Life Sciences, University of Nottingham, Nottingham, UK; 12 Smurfit Institute of Genetics, Trinity College Dublin, Dublin, Ireland; 13 UCD Conway Institute of Biomolecular and Biomedical Research, University College Dublin, Dublin, Ireland; 14 UCD One Health Centre, University College Dublin, Dublin, Ireland

**Keywords:** Cattle, Genome-enabled Breeding, Imputation, Low-Coverage Whole Genome Sequencing

## Abstract

**Background:**

In cattle, most commercial single-nucleotide polymorphism (SNP) genotyping arrays have been shown to be suboptimal for capturing genomic variation in non-European populations, particularly in African cattle. Low-coverage whole-genome sequencing (LCWGS) followed by imputation provides a cost-effective method for genotyping that is more adaptable and can outperform genotyping arrays.

**Results:**

Here, we generate a high-quality reference imputation panel representative of the complex ancestries of cattle populations in Africa to enable the deployment of LCWGS. To do so, we generated 116 high-coverage (between 20‒24×) new African cattle genomes, representing most cattle breeds across the continent. We combined this data with publicly available genomes from other regions to build a reference panel that comprised over 3,300 cattle genomes from 133 cattle populations, thus capturing the genetic diversity of domestic cattle across the world. After applying a high filtering step to remove poor genome sequences with very low sequence coverage, we retained 1,882 with an average coverage of 7×. We show that the imputation pipeline implemented, based on this reference panel, provides highly accurate genotypes of common (> 99% accuracy) and rare (> 98% accuracy) variants in genome coverage as low as 0.5×.

**Conclusion:**

This panel provides an important new resource for genetic improvement and conservation of African cattle populations.

**Supplementary Information:**

The online version contains supplementary material available at 10.1186/s12864-026-12818-4.

## Background

Genome-enabled breeding routinely uses genome-wide single-nucleotide polymorphisms (SNPs) to estimate the breeding value of individuals, and has been successfully applied to increase productivity in multiple species of livestock [1–3] and crop plants [4, 5]. Such approaches have increasingly relied on commercial SNP genotyping arrays, which enable efficient genotyping of thousands of pre-defined SNPs that were previously identified by whole-genome sequencing (WGS).

In cattle, multiple SNP array platforms exist that cover between ~ 3,000 and ~ 800,000 SNPs. Unfortunately, however, most of the SNPs represented in these arrays were originally discovered in European taurine cattle populations and have been shown to be suboptimal for capturing genomic variation in other populations, particularly in African cattle [6]. The low sensitivity of these pre-designed arrays reflects the dynamic evolutionary history of cattle in Africa, which possess a diverse genomic makeup that has been influenced by over 6,000 years of adaptation to a myriad of environments [7], episodic gene flow from local aurochs [8], and substantial gene flow from indicine cattle [9].

Africa accounts for 25% of the global livestock population, and the livestock industry employs a large labour force on the continent [10]. Livestock represent a significant contributor to the agricultural gross domestic product (GDP) in many African countries (between 35% and 80%) [11, 12]. Yet most African cattle breeds have not been subject to scientifically-informed breeding practices for enhanced productivity, and as a result they are much less productive than their European counterparts. While European breeds might offer benefits to smallholder African farmers in the short term, their sensitivity to climatic conditions and lack of resistance to local diseases mean that they are a risky investment in the long term. This is particularly problematic as cattle often serve as mobile capital in many farming communities in sub-Saharan Africa [13]. Therefore, improving the productivity of local breeds, which are well adapted to local environmental constraints, through the introduction of new breeding techniques, such as genome-enabled breeding provides an attractive solution.

Low-coverage whole-genome sequencing (LCWGS) followed by imputation provides a cost-effective method for genotyping that is more adaptable and can outperform SNP genotyping arrays [14–17]. This technique (LCWGS) involves imputation of SNPs from low-coverage WGS data using large reference panels, and it offers multiple advantages compared to traditional SNP arrays. Firstly, LCWGS allows for the genotyping of many more variants because it is not just limited to the SNPs represented on an array. Secondly, LCWGS does not require complex and costly pre-selection of variants and probe design which are necessary to develop SNP arrays. Lastly, LCWGS provides a longer-term solution as imputation can be conducted using any pre-defined set of SNPs from any large reference panel of a similar ancestral background [18]. These advantages make LCWGS a more flexible and cost-effective technology than SNP arrays for genotyping millions of variants in less studied populations.

Here, we aim to generate a high-quality reference imputation panel representative of the complex ancestries of cattle populations in Africa to enable the deployment of LCWGS. To do so, we present a novel library building protocol, which allows for significant savings compared to traditional protocols. We used this protocol to generate 116 high-coverage (between 20‒24×) new African cattle genomes, representing most cattle ancestries across the continent. We combined these data with publicly available genomes from other regions to build a reference panel that comprises over 3,300 cattle genomes from 133 cattle populations and captures the genetic diversity of cattle across the world. We show that the imputation pipeline implemented, based on this reference panel, provides highly accurate genotypes of common (> 99% accuracy) and rare (> 98% accuracy) variants with genome coverage as low as 0.5×. This cost-effective LCGWS panel provides an important novel resource enabling researchers to study and improve the conservation, efficiency, and resilience of African cattle populations.

## Results

### Overview of the reference panel

**Fig. 1:**
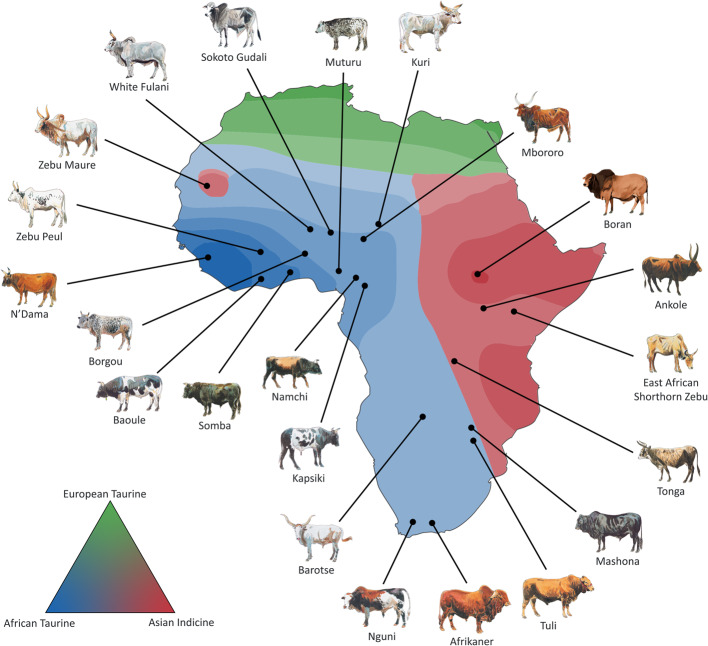
Cattle ancestry gradient across Africa computed using ADMIXTURE (*K* = 3) and projected on a map using tess3. Cattle art images [19, 20]

We generated over 40 billion reads for 116 African cattle, with an average depth of coverage of 24.4×. These data were merged with additional publicly available genomic data to build a reference panel of 3,399 animals representing 133 cattle breeds. The reference panel comprised *B. taurus* (2,243 cattle), *B. indicus* (618 cattle), and taurine × indicine hybrids (538 cattle). A spatial representation of the ancestry coefficient of the African breeds are shown in Fig. 1. A reference panel was generated using 1,882 genomes (Supplementary Table 1), which contained 151,612,424 SNPs after filtering for a minimum of 7× sequencing depth. Further filtering was used to remove multiallelic SNPs, which left 89,974,216 biallelic SNPs for imputation. We obtained a highly accurate phased panel for common and rare variants using SHAPEIT5 [21]. The phased reference panel was used for GLIMPSE imputation. A summary of this workflow is depicted in Supplementary Fig. 1.

### Imputation accuracies across cattle population

**Fig. 2:**
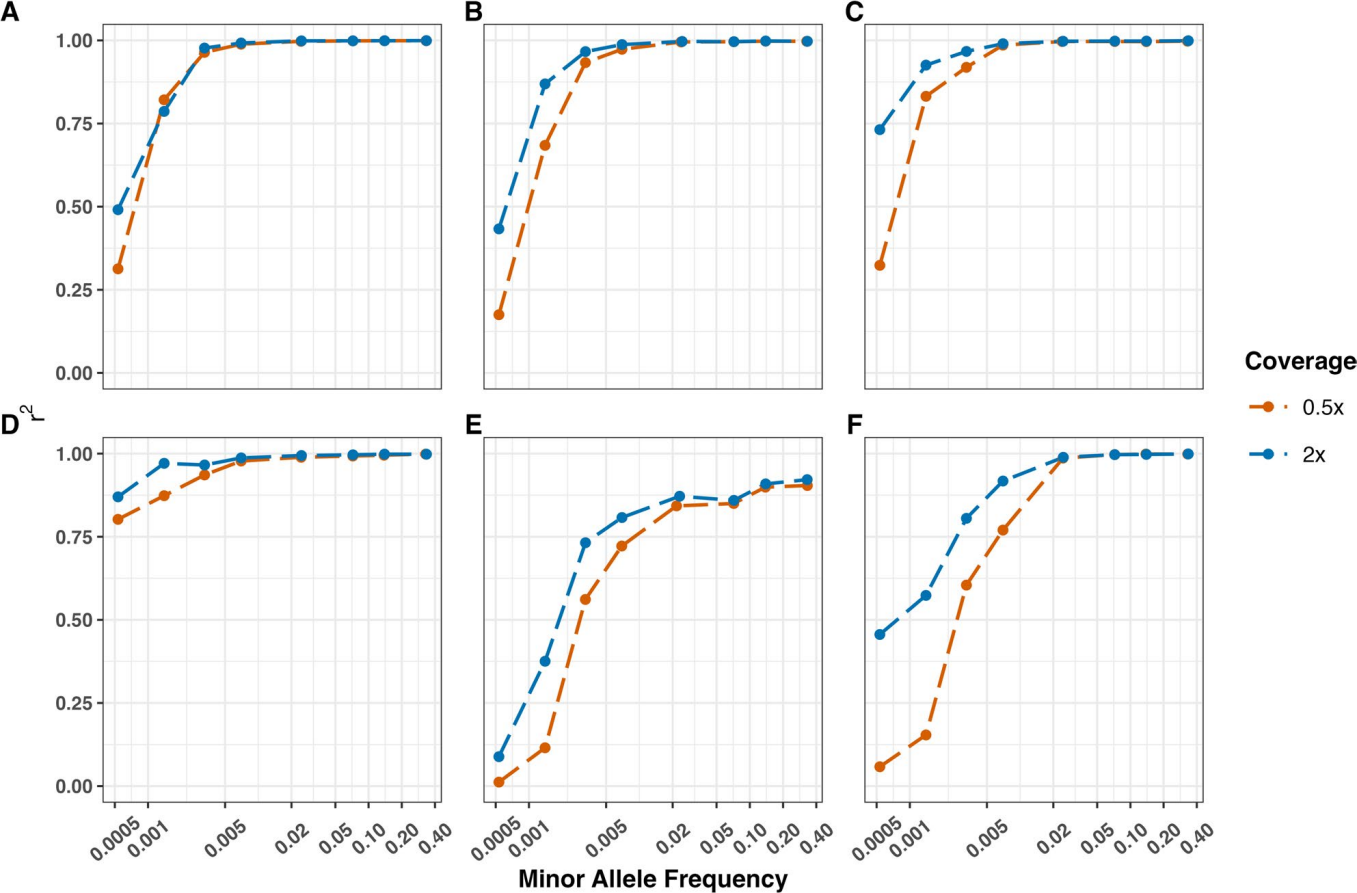
Evaluation of imputation accuracy (squared Pearson correlation coefficient *r*^2^) across MAF values and INFO score of 0.95 for genomes downsampled to a depth of coverage of 0.5× and 2× in six cattle breeds. **A** Afrikaner (African taurine x indicine, South Africa), (**B**) Kilimanjaro Zebu (indicine, East Africa), (**C**) Kuri (taurine, West Africa), (**D**) Criollo (taurine, South America), (**E**) Hariana (zebu, South Asia), and (**F**) Shorthorn (taurine, UK)

Imputation accuracies for African cattle breeds ranged from 92% to 98% for 0.5× sequencing depth. The Afrikaner, Kilimanjaro and Kuri breeds showed an increase in imputation accuracy measured by the Pearson correlation (*r*^2^) between minor allele frequency (MAF) values of 0.001 and 0.005 and then plateaued for the filtered INFO score cutoff of 0.95 at 0.5× sequencing depth (Fig. 2). The filtered INFO score refers to a threshold of quality metrics used to filter imputed variants based on the posterior genotype probability (GP). A high cutoff of 0.95 applied to imputed variants yields high-confidence variants. In contrast, the non-filtered INFO score relates to the quality metrics of all imputed variants without applying any filters. We observed that the non-filtered INFO score exhibited slightly lower imputation accuracies than the filtered INFO score. In the non-filtered INFO score, the accuracy increased to 95% for MAF values between 0.001 and 0.020 in the Afrikaner breed, after which it plateaued. The Kuri and Kilimanjaro breeds plateaued at 85% and 90% accuracy for MAF greater than 0.050 at 0.5× coverage (Fig. 2 and Supplementary Fig. 2).

Imputation accuracy of rarer alleles increased notably with sequencing depths (Fig. 2 and Supplementary Figs. 2‒5). For the African breeds, imputation accuracy steadily improved from a MAF greater than 0.001 and reached a plateau at 99% imputation accuracy for a MAF of 0.005 for both filtered and non-filtered INFO cutoffs at 2× coverage (Fig. 2 and Supplementary Fig. 4). This trend was also observed at 1× coverage (as shown in Supplementary Fig. 3). Additionally, the imputation accuracy exhibited a further increase from a MAF of 0.0005 and plateaued at 99% imputation accuracy from a MAF of 0.001 at 4× sequencing depth in all African breeds (see Supplementary Fig. 5).

**Fig. 3:**
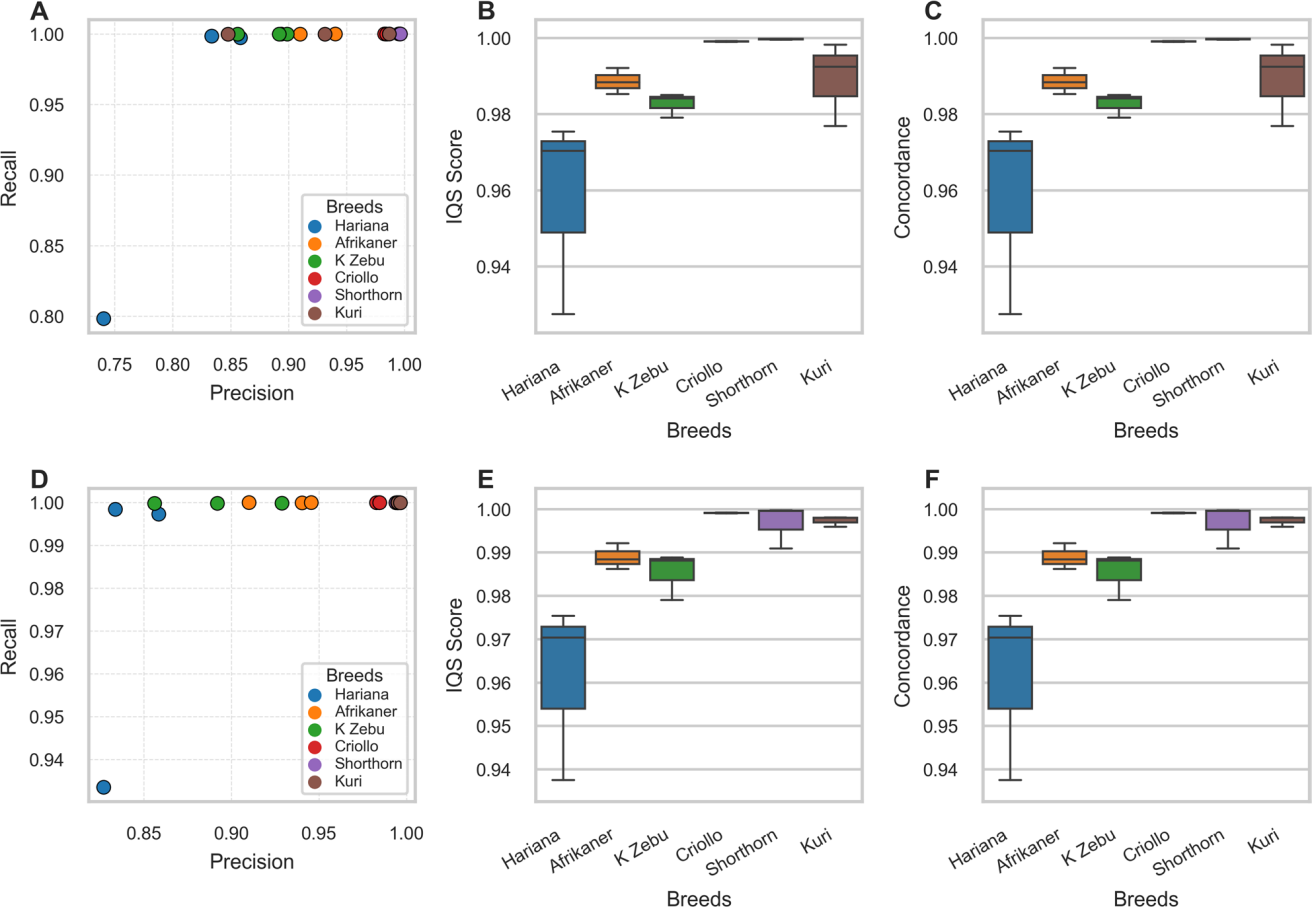
Summary of imputation accuracy statistics for 0.5× and 2× coverage with a MAF > 0.05. The top row shows the (**A**) recall imputation accuracy, (**B**) imputation quality score (IQS), and (**C**) the concordance statistics for 0.5× coverage and MAF > 0.05. The bottom row shows the (**D**) recall imputation accuracy, (**E**) imputation quality score (IQS), and (**F**) the concordance statistics for 2× coverage and MAF > 0.05. Breed code Kilimanjaro Zebu (K Zebu)

The Imputation Quality Score (IQS) and concordance statistics of the imputed genotypes with a MAF of 0.05 indicate greater than 97% imputation accuracies for the African breeds, the Criollo breed, and the European breed (Fig. 3). For the Asian indicine breed (Hariana), we achieved IQS and concordance accuracy rate greater than 90% at 0.5× coverage. IQS and concordance assessment statistics for 2× coverage at a MAF of 0.05 show over 98% accuracies for African breeds, Criollo, and European cattle (Fig. 3). Precision ranged from 85% to 99% with a 99% recall rate. Hariana cattle demonstrated an IQS and concordance accuracy rate exceeding 94% (Fig. 3).

**Fig. 4:**
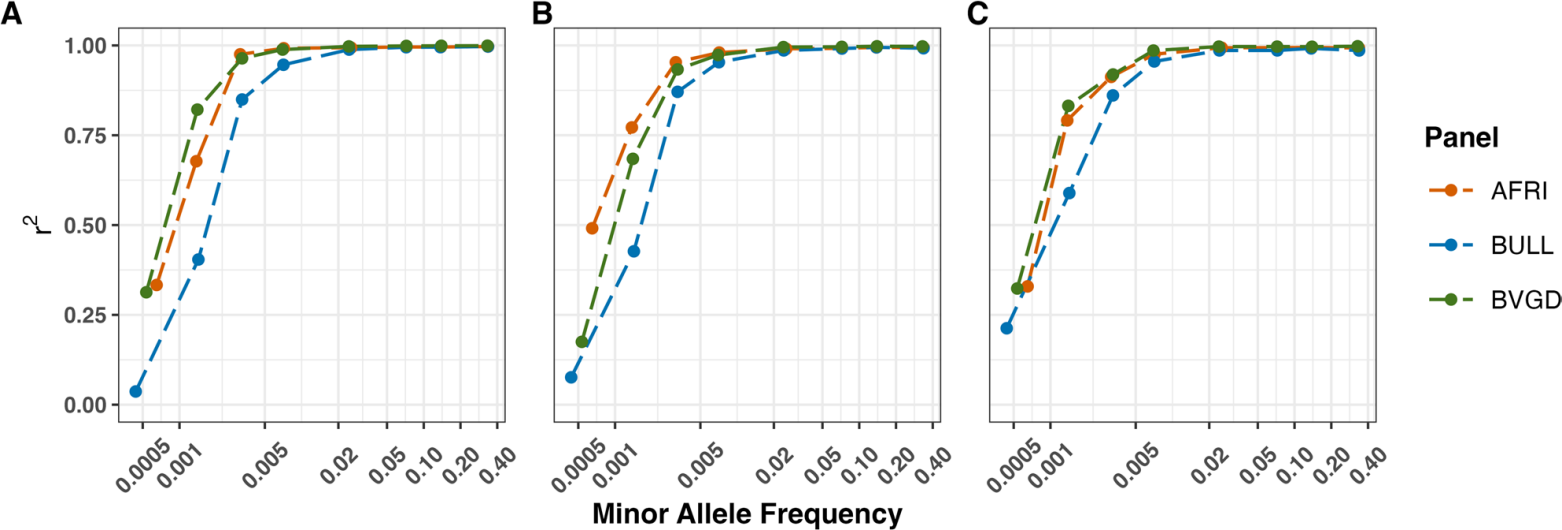
Assessing imputation accuracy for 0.5× coverage on chromosome 19 in three African breeds using high-quality reference panels at various MAF values. Evaluation of accuracy measured by squared Pearson correlation coefficient *r*^2^ in the (**A**) Afrikaner (African taurine x indicine, South Africa), (**B**) Kilimanjaro Zebu (indicine, East Africa), and (**C**) Kuri (taurine, West Africa). The reference panel is AFRI (our study – 1,882 cattle), BULL (Run 8 of the 1000 Bull Genomes Project – 1842 cattle), and BVGD (the Bovine Variation Genome Database – 2976 cattle)

We evaluated the performance of our African reference panel (AFRI) alongside two other major reference panels: the Bovine Genome Database panel (BGVD) and Run 8 of the 1000 Bull Genome Project (BULL). Our results show that our imputation reference panel for the African cattle outperformed both the BGVD and BULL panels in terms of imputation accuracy. For the African breeds (Afrikaner, Kilimanjaro zebu, and Kuri), imputation accuracy steadily improved from a MAF greater than 0.001 and reached a plateau of 99% imputation accuracy at a MAF of 0.005 for our panel (AFRI). We observed a slightly similar result with BGVD, with imputation accuracy plateauing at 97%, while the BULL reached a plateau at 90% imputation accuracy for a MAF of 0.005, as demonstrated in Fig. 4. We compared the total number of imputed SNPs between our AFRI and BVGD panels, and our panel outperformed BVGD, achieving a more accurate total number of SNPs imputed across MAF (Supplementary Fig. 6).

## Discussion

Our new imputation panel offers a powerful tool to generate high-quality genotypes (> 99% accuracy) for approximately 18 million SNPs (with MAF of 0.05 and higher) based on only 0.5× sequencing depth in a wide range of African cattle populations. In comparison with the large reference panels from the 1000 Bull Genomes Project (BULL) and the Bovine Variation Genome Database (BVGD), our panel demonstrated better performance with high imputation accuracy for African indicine cattle (Kilimanjaro Zebu) while showing similar imputation accuracy for taurine and admixed cattle (Kuri and Afrikaner) (Fig. 4). The imputation accuracy of the BULL reference panel was lower than the other two panels, which is likely due to the reduced representation of African cattle ancestries in this panel. A comparison with the BVGD panel shows that our panel captures substantially more imputed SNPs across MAF bands (Supplementary Fig. 6).

Our imputation reference panel accuracies represent a substantial improvement over previous studies that benchmarked the imputation of large reference panels (BULL and BVGD) and high-density SNP arrays (777 K and 649 K) in parallel with several other lower-density SNP arrays [6, 22–24]. This previously low imputation accuracy (~ 60% at MAF of 0.05) is likely due to the ascertainment bias of commercial array SNPs towards European populations, specifically in medium to high-density SNP arrays [6, 22].

Higher levels of imputation accuracy (~ 80% at MAF of 0.05) using a high-density SNP array have been reported in East African crossbred dairy cattle [22]. The development of this crossbred dairy breed was achieved through unsystematic crossbreeding between indigenous cattle and exotic dairy breeds such as Ayrshire, Friesian, Holstein and Jersey [25]. Genotyping has revealed that a significant number of crossbred dairy cattle have more than 50% ancestry from exotic breeds [25] and higher levels of high-density SNP array accuracy in these breeds compared to other populations in Africa is due to their European ancestry.

While 0.5× sequence depth provided sufficient accuracy for low- and medium-frequency variants (MAF 0.01‒0.05), we observed a significant improvement in imputation accuracy for lower frequency variants (0.001‒0.005) when sequencing depth reached 2× (Fig. 2). Therefore, for projects focusing on demographic reconstruction or inbreeding estimation, which rely on lower-frequency variants, we recommend sequencing at 2× depth.

Our results also show that the ancestry composition of the reference panel plays a key role in imputation accuracy. For Asian breeds with almost 100% indicine ancestry, such as the Hariana, we show that the imputation accuracy plateaued at ~ 80%, even when considering medium-frequency variants (MAF > 0.10, with no INFO cutoff) at 4× sequencing depth (Supplementary Fig. 5). The performance of the Hariana cattle breed imputation plateaued at 95% (MAF > 0.10, with INFO cutoff) at 4× coverage (Supplementary Fig. 5). Given that imputation relies heavily on the genetic relationship between target and reference populations, the current reference panels limit performance for Asian indicine cattle. Expanding the collection of sequence data from South Asian breeds is therefore essential to improve panel representativeness and capture global genetic diversity.

In our laboratory, based on current costs for consumables for DNA extraction, library build, and sequencing, 0.5× and 2× cattle genomes cost ~$20 and ~$40, respectively. This represents a major cost advantage over whole-genome sequencing at 10× (~$140), and traditional SNP genotyping arrays (~$55‒$155). In addition, SNP arrays have limitations, both in the number of SNPs they target (3–777 K SNPs compared to > 18 M SNPs here) and an inherent bias towards SNP content representative of European cattle breeds. Therefore, we forecast that the steadily decreasing cost of high-throughput sequencing will significantly impact the livestock industry in lower-income countries, particularly in Africa, where livestock production contributes between 35 and 80% of GDP [12].

## Materials and methods

### Sample collection, genome sequencing, and publicly available genome data

The primary dataset used for this analysis consists of both newly generated, previously unpublished, and publicly available cattle genome sequences. Here we generated and obtained a total of 116 genomes from 32 populations of cattle: Afrikaner, Arado, Baoule, Barotse, Blonde d’Oulmès-Zaër, Bootse, Borgou, Danakil, Djakore, IringaRed, Kapsiki, Kavirondo Zebu, Kuri, Lagune, Mashona, Mbororo, N’Dama (Guinea), N’D’ama (Gambia), N’Dama (Nigeria), N’Dama (Senegal), Namchi, Nguni, Raya Zebu, Sokoto Gudali, Somba, Tonga, Tuli, Watusi, White Fulani, Zebu Kilimanjaro, Zebu Malawi, and Zebu Peul. The blood samples from these animals were collected, and the DNA extracted, and purified as previously described by MacHugh et al. [26], Hanotte et al. [27], Troy et al. [28] and Freeman et al. [29]. These 116 genomes also included data from raw fastq files for 11 unpublished cattle genomes that were obtained from Acceligen.

Sequencing libraries were prepared with a downscaled version of the bead-based tagmentation kit from Illumina (DNA-prep, Illumina San Diego, USA) following the manufacturer’s protocol with several adjustments. A volume of 3 µl containing 200 to 500 ng of genomic DNA was mixed with 2 µl of tagmentation mastermix (DNA Prep kit, Illumina), incubated at 55 °C for 15 min and then for a further 15 min at 37 °C after addition of Tagmentation stop buffer (DNA Prep kit, Illumina). Tagmented DNA bound to magnetic beads was then purified according to the kit protocol. The beads were then added to a PCR mix containing unique double index (UDI) primers (xGen™ UDI, Integrated DNA technologies) and amplified for 7‒9 cycles. Products were purified with SPRI beads (DNA Prep kit, Illumina). Concentration was measured with 50 µl of Qubit HS reagent on a plate-reader (Fluostar Omega, BMG Labtech, Ortenberg, Germany) and equal amounts of the library were pooled for sequencing. Libraries were then sequenced on a NovaSeq S4 platform (Illumina) at a minimum 20× sequencing depth.

We then downloaded raw reads from 3,283 publicly available cattle genomes from the European Nucleotide Archive (ENA) (Supplementary Table 2), which were merged with the 116 new genomes, totalling 3,399 global cattle genomes. Our dataset then comprised *B. taurus* (2,243 cattle), *B. indicus* (618 cattle), and taurine × indicine hybrids (538 cattle).

### Data processing

Sequence data were processed using the Genome Analysis Tool Kit (GATK) v.4.1.9.0 best practices [30]. First, we evaluated per-base sequence quality for all raw reads using FastQC v.0.11.9 [31] and mapped reads against the bovine reference genome (ARS-UCD1.2) [32] using bwa-mem2 v.2.1 [33] with default parameters. The Picard tool v.2.24.1 was used to sort the bam files and create index files [34]. PCR duplicates were identified using ‘MarkDuplicatesSpark’ in GATK [35], and ‘BaseRecalibrator’ and ‘ApplyBQSR’ of GATK were used to limit the retention of sequencing errors in our dataset. These analyses were based on known variants provided by the 1000 Bulls Genomes Project (ARS1.2PlusY_BQSR_v3.vcf.gz; www.1000bullgenomes.com*).* Base qualities were examined before and after BQSR reports using ‘Analyze covariate’ for the initial and post-recalibration tables to assess whether base qualities were corrected as expected.

Raw GVCFs were generated using ‘HaplotypeCaller’ in GATK with the ‘-ERC GVCF’ option. We created a genomic database for all the individual GVCFs using ‘GenomicsDBimport’ and created joint variant calling using the ‘GenotypeGVCFs’ option. We evaluated the quality of the joint variant file by computing Variant Quality Score recalibration with default hard filters using GATK tools [30]. Multiple resources were used for exploring variant quality and filtering options: 1) dbSNPs from Ensembl; 2) a vcf file obtained from the 1000 Bull Genomes Project; and 3) two high-density SNP array (BovineHD 777K) datasets comprising a representative sample of global cattle populations [6, 20]. We also filtered minimum read depth and genotype quality score ‘FMT/DP < 5 && FMT/GQ < 20’ and removed multiallelic SNPs and indels using bcftools [36].

### Imputation workflow

#### Reference panel

The imputation pipeline was implemented in snakemake (v.5.16) [37] with the R environment (v.4.1) [38] and ggplot2 [39] was used for statistical analyses and plotting. Briefly, to build a reference panel, we first genotyped 3,399 cattle genomes, including 116 novel genomes, using GATK best practices. Only samples with > 7× depth of sequencing coverage were included in the reference VCF reference panel used for imputation (1,882 out of 3,399 genomes).

#### Phasing

We used SHAPEIT5 v.5.1.0 21 for phasing. We first split the panel into chromosomes and phased each chromosome separately using SHAPEIT5 [21]. We used two steps for this process in SHAPEIT5: for Step 1, we chunked each chromosome into 20 cM pieces and phased common variants (with a minor allele frequency ≥ 0.1%) using SHAPEIT5_phase_common. We then ligated the phased common variants using SHAPEIT5_ligate. For Step 2), we used the ligated variants as input for the second phasing step of the rare variants (with a minor allele frequency < 0.1%) using SHAPEIT5_rare. The fully phased chunks were concatenated, and multiallelic variants were removed using bcftools [40].

### Downsampling for imputation benchmarking

To assess our imputation reference panel, we selected six representative populations to capture global genomic diversity (Hariana [South Asia], Afrikaner [South Africa], Shorthorn [Europe], Kilimanjaro zebu [East Africa], Criollo [South America], and Kuri [West Africa]). A total of 18 animals from six cattle breeds were downsampled to various depths for imputation benchmarking. First, we removed these animals from the reference panel. We used the bcftools +fill-tags flag to re-compute fields after sample removal and filtered the missing variants with the F_missing flag field [40]. We then used bcftools to extract site positions from the reference panel and computed genotype likelihoods on those sites from high-coverage BAM files. The genotype likelihoods for the animals used for benchmarking were computed using bcftools ‘mpileup’ and ‘call -Aim -C alleles’ (to only genotype alleles present in the VCF panel). We then used samtools to downsample the bam files of these animals to specific coverage: [0.5, 1, 2, and 4×]. Following this, bcftools was used to extract site positions from the downsampled bam files. Finally, we used bcftools mpileup -I -E -a ‘FORMAT/DP’ and ‘call -Aim -C’ alleles to estimate genotype likelihood in each downsampled bam file.

### Imputation

We used GLIMPSE v.1.1.1 [41] for imputation by first splitting the extracted site position of the reference panel with the GLIMPSE chunk tool with default settings of --window-size 2,000,000 and --buffer-size 200,000 to improve computational efficiency. We then used the downsampled genotype likelihoods, site positions of the reference panel, a genetic map and imputation chunks with their buffers described above as input for the imputation step using the GLIMPSE_phase tool. Following this, we merged the imputed chunks for each animal sample using the GLIMPSE_ligate tool and phased the ligated imputed vcf file with GLIMPSE_sample.

### Imputation accuracy

Imputation accuracy was measured as genotyping concordance between high coverage genotyped calls and imputed genotype was estimated for different INFO score cutoffs (0.00, 0.80, 0.90, and 0.95), MAF values (0.001‒0.500) and coverages (0.5, 1, 2, and 4×) using a Pearson correlation (*r*^2^; see Fig. 2) as implemented in the GLIMPSE_concordance tool. We also computed additional summary statistics [42]. Firstly, we calculated the Imputation Quality Score (IQS), which computes the difference between imputed genotype probabilities to true genotypes and is complementary to *r*^2^ [42]. IQS can help to detect false positives due to chance agreement [43]. Then, we also computed the imputation concordance rate [42], which is the correlation of correctly inferred genotypes to all the imputed genotypes.

### Assessing the performance of large reference panels

We compared our panel with a recently designed large reference panel from the Bovine Genome Variation Database (BGVD), which includes 2,976 cattle from around the world [23], and Run 8 of the 1000 Bull Genomes Project (BULL) [44] constituting 1,842 global cattle. We evaluated the accuracy of imputation for 0.5× coverage in three African cattle breeds (Afrikaner, Kuri, and Kilimanjaro Zebu) using the detailed GLIMPSE pipeline.

### Interpolated map of African cattle genomic ancestry

Ancestry coefficients for African cattle breeds were computed using ADMIXTURE [45] with *K* = 3, which represents pure European taurine, pure African taurine, and indicine ancestry (Pakistan *B. indicus*) components (Supplementary Table 3). The resulting *q* matrix and cattle individual geographic location were then used as input for tess3 [46]. A spatial representation of ancestry coefficients was then generated for the cattle breeds and displayed in Fig. 1.

## Supplementary Information

Supplementary Material 1.

## Data Availability

The data generated from 116 African cattle for this study is available from the NCBI sequence read archive (SRA) database (BioProject ID: PRJNA1170881).

## Abbreviations

SNP: Single-Nucleotide Polymorphism
LCWGS: Low-Coverage Whole-Genome Sequencing
WGS: Whole-Genome Sequencing
GDP: Gross Domestic Product
GP: Genotype Probability
IQS: Imputation Quality Score
MAF: Minor Allele Frequency
AFRI: African Reference panel
BGVD: Bovine Genome Database
BULL: Bull Genome Project
ENA: European Nucleotide Archive
GATK: Genome Analysis Tool Kit
