# Supplementary material for: Accurate imputation of African cattle genomes using a diverse reference panel
Source: BMC Genomics. 2026 May 6;27:452. doi: 10.1186/s12864-026-12818-4 (PMC13154541; doi:10.1186/s12864-026-12818-4)
Supplement: Supplementary file 1 — Supplementary Material 1. [file 12864_2026_12818_MOESM1_ESM.docx]

Supplementary: Accurate Imputation of African Cattle Genomes Using a Diverse Reference Panel

Said I. Ng’ang’a ^1,2,3*^, James A. Ward ^4,5^, Stephen J. Rossiter ^1^, Chris G. Faulkes ^1^, Katia Bougiouri ^6^, Gillian P. McHugo ^4^, Fenton P. D. Cotterill ^7^, Atunga Nyachieo ^3^, Olaf Thalmann ^2^, Ivica Medugorac^5^, Stefan Krebs ^8^, Tad S. Sonstegard ^9^, Olivier Hanotte ^10,11^, Daniel G. Bradley ^12^, Gary Vaughan-Smith ^7^, David E. MacHugh ^4,13,14*^, Laurent A.F. Frantz ^1,2,15*^

^1^School of Biological and Behavioural Sciences, Queen Mary University of London, London, United Kingdom

^2^Palaeogenomics Group, Department of Veterinary Sciences, Ludwig Maximilian University, Munich, Germany

^3^Bioinformatics and Health Informatics Group, Kenya Institute of Primate Research, Nairobi, Kenya

^4^Animal Genomics Laboratory, UCD School of Agriculture and Food Science, University College Dublin, Dublin, Ireland

^5^Population Genomics Group, Department of Veterinary Sciences, Faculty of Veterinary Medicine, Ludwig Maximilian University of Munich, Munich, Germany

^6^Section for Molecular Ecology and Evolution, Globe Institute, University of Copenhagen, Copenhagen, Denmark

^7^SilverStreet Capital, London, United Kingdom

^8^Laboratory for Functional Genome Analysis, Gene Center, Ludwig Maximilian University of Munich, Munich, Germany

^9^Acceligen, Eagan, MN USA

^10^International Livestock Research Institute, Addis Ababa, Ethiopia

^11^School of Life Sciences, University of Nottingham, Nottingham, United Kingdom

^12^Smurfit Institute of Genetics, Trinity College Dublin, Dublin, Ireland

^13^UCD Conway Institute of Biomolecular and Biomedical Research, University College Dublin, Dublin, Ireland

^14^ UCD One Health Centre, University College Dublin, Dublin, Ireland

^15^ Lead contact

^*^ Corresponding authors: Said I. Ng’ang’a: [s.i.nganga@qmul.ac.uk](mailto:s.i.nganga@qmul.ac.uk); David E. MacHugh [david.machugh@ucd.ie](mailto:david.machugh@ucd.ie); Laurent A.F Frantz: [laurent.frantz@lmu.de](mailto:laurent.frantz@lmu.de)

**
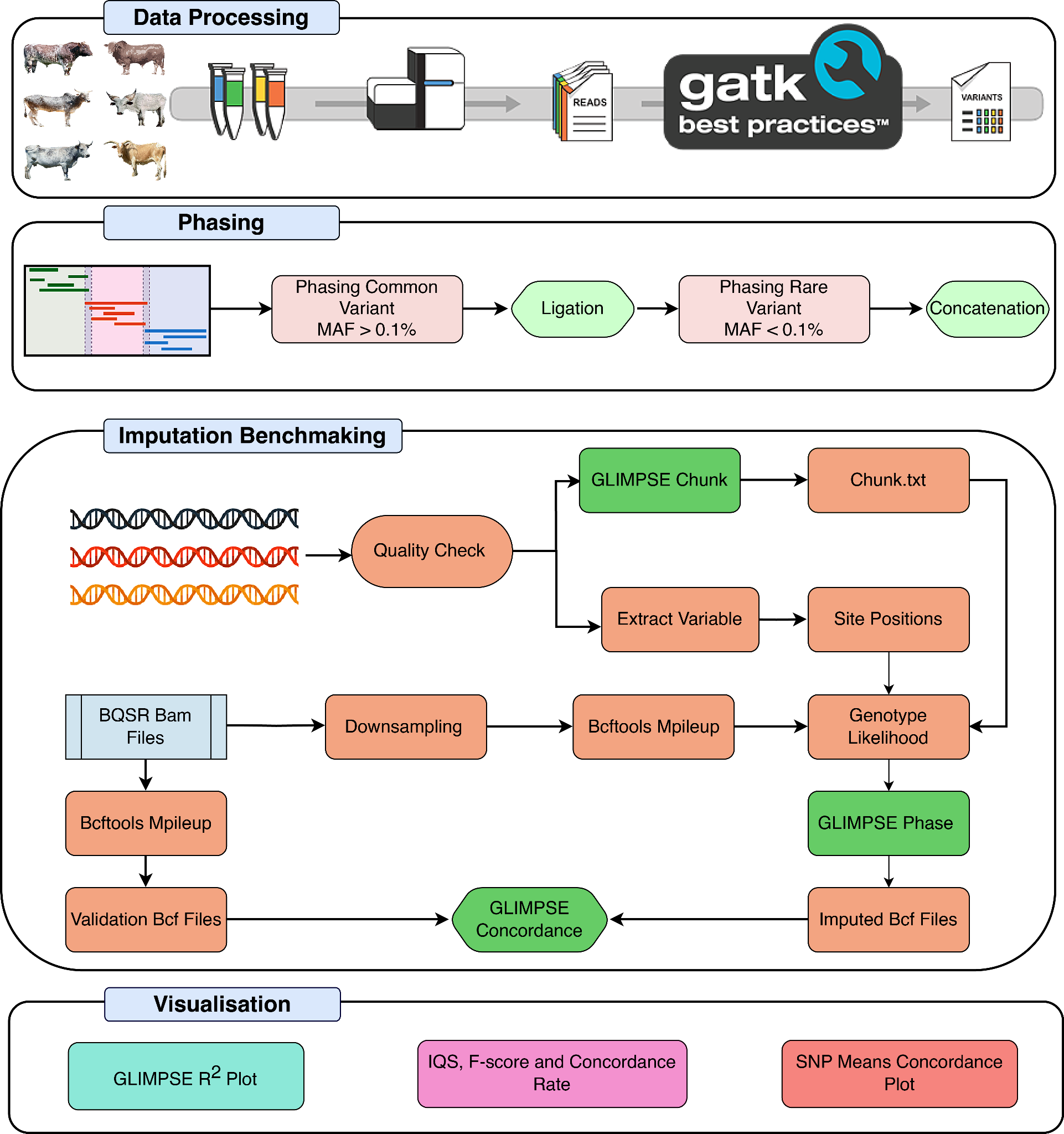
**

**Supplementary Figure 1**: Evaluation of imputation accuracy (squared Pearson correlation coefficient *r*^2^) across MAF values and INFO scores for genomes downsampled to a sequencing depth of 0.5× in six different cattle breeds. Afrikaner (taurine, South Africa), Kilimanjaro Zebu (indicine, East Africa), Kuri (taurine, West Africa) Shorthorn (taurine, UK), Hariana (indicine, South Asia), and Creole (taurine, South America).


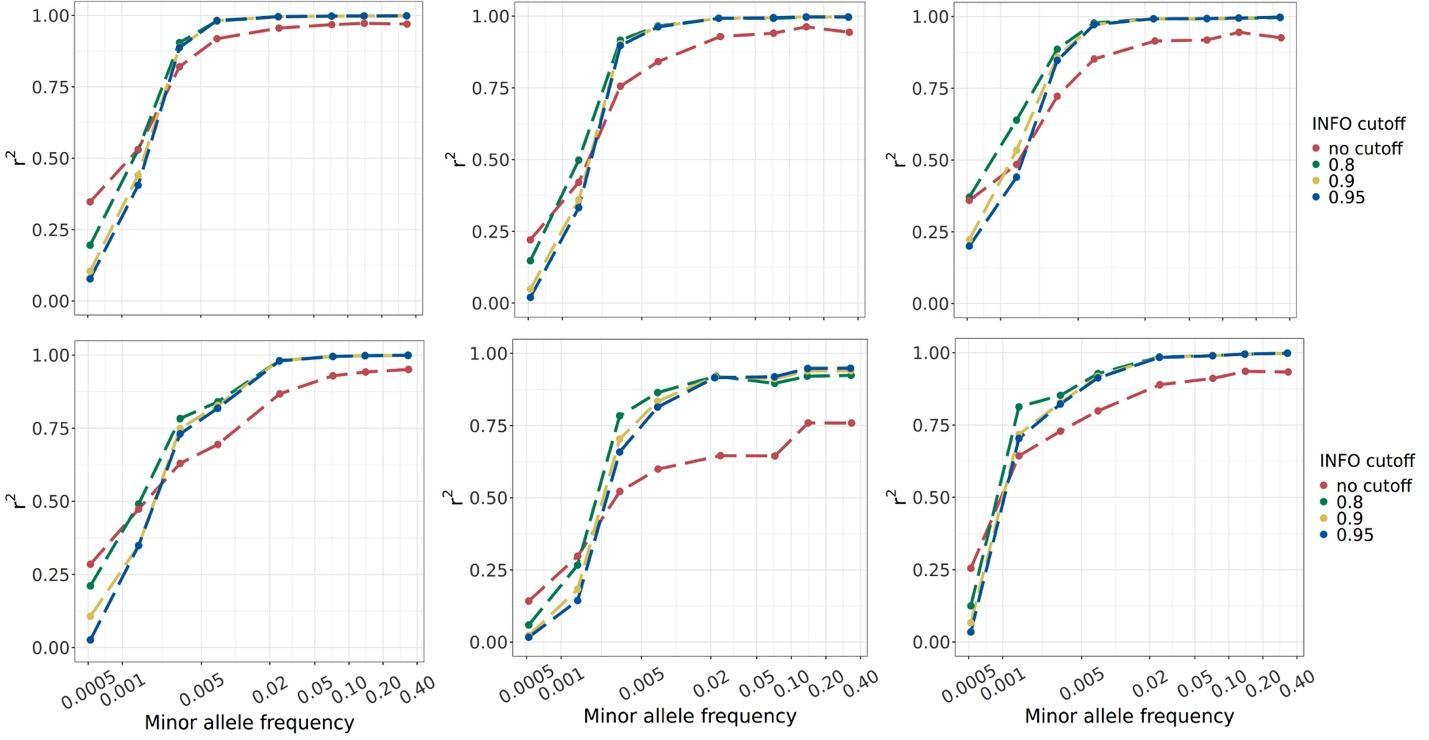


**Supplementary Figure 2**: Evaluation of imputation accuracy (squared Pearson correlation coefficient *r*^2^) across MAF values and INFO scores for genomes downsampled to a sequencing depth of 0.5× in six different cattle breeds. Afrikaner (taurine, South Africa), Kilimanjaro Zebu (indicine, East Africa), Kuri (taurine, West Africa) Shorthorn (taurine, UK), Hariana (indicine, South Asia), and Creole (taurine, South America).


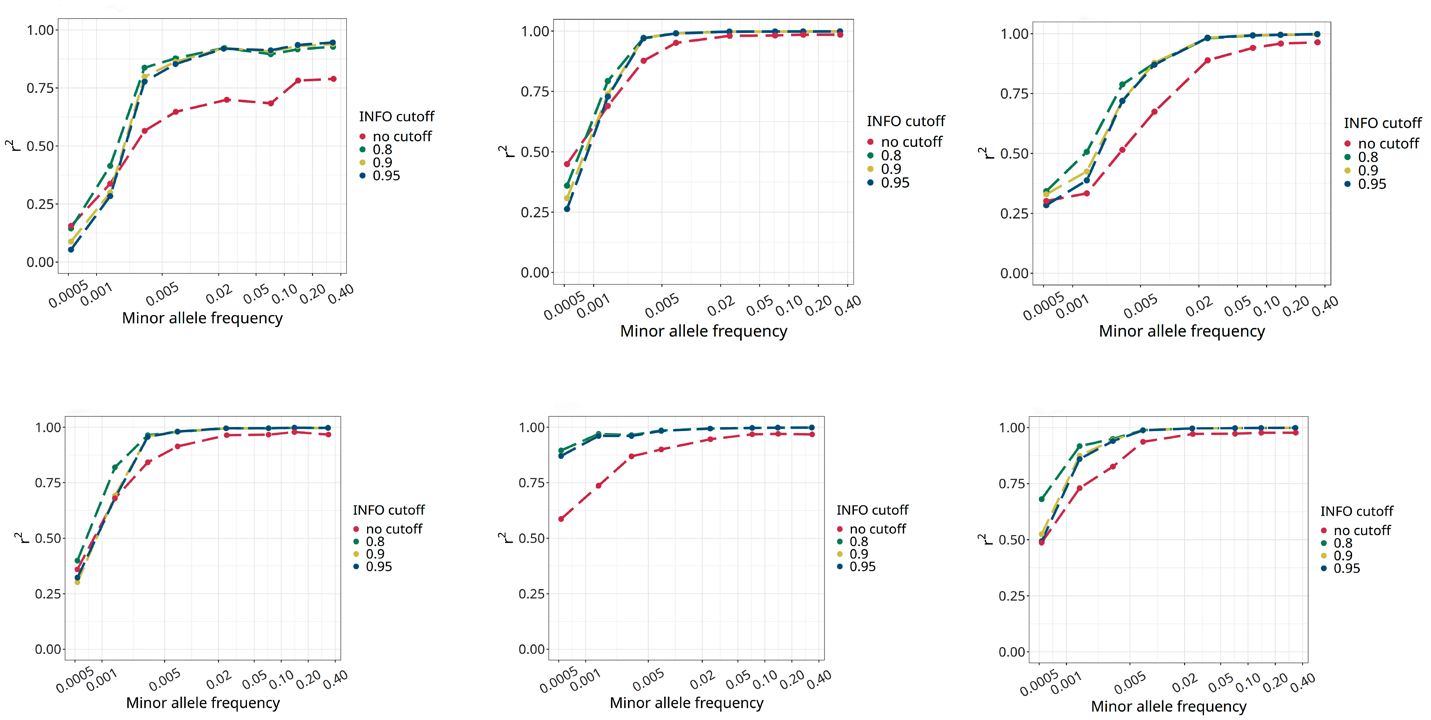


**Supplementary Figure 3**: Evaluation of imputation accuracies and their distributions across MAF, *r*^2^ and INFO scores. Six different cattle breeds are shown that were assessed for imputation accuracy at 1× sequencing depth (Hariana [South Asia], Afrikaner [South Africa], Shorthorn [Europe], Kilimanjaro Zebu [East Africa], Creole [South America], and Kuri [West Africa]).


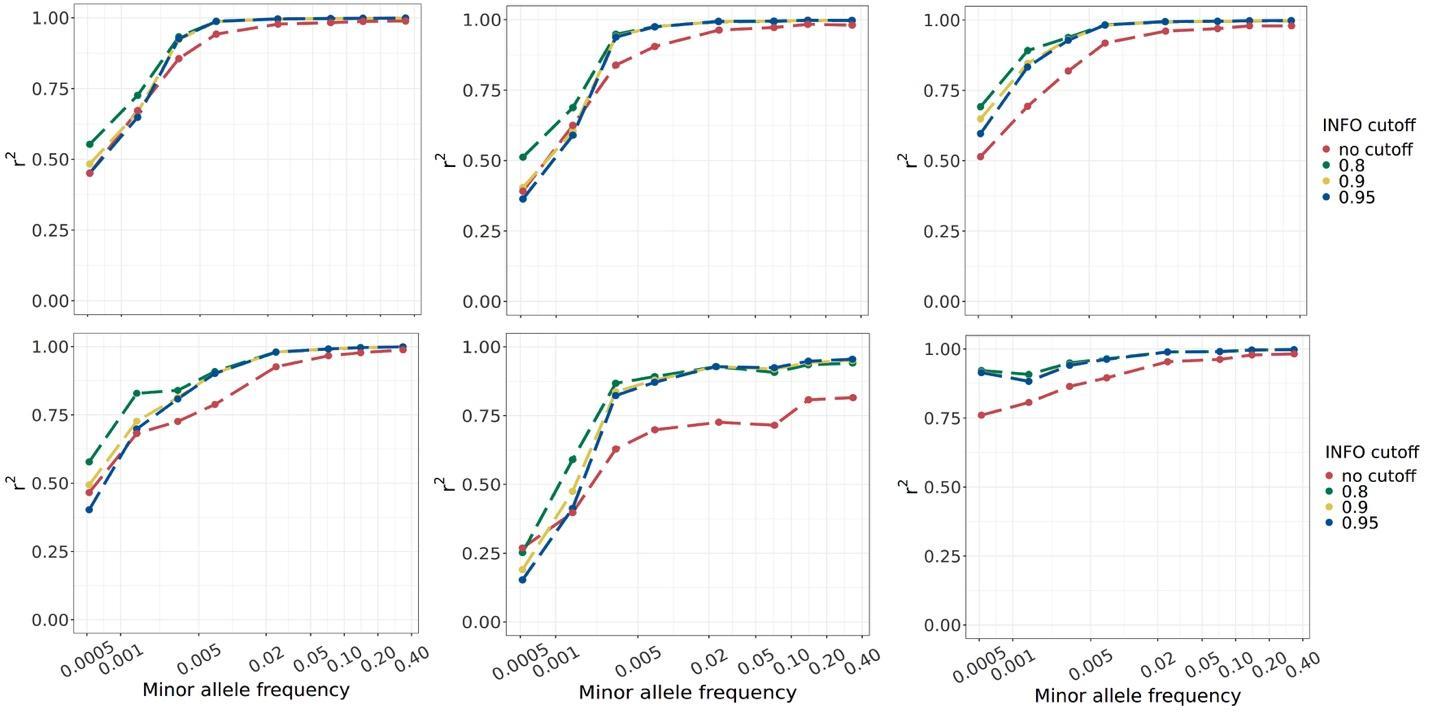


**Supplementary Figure 4**: Evaluation of imputation accuracy (squared Pearson correlation coefficient *r*^2^) across MAF values and INFO scores for genomes downsampled to a sequencing depth of 2× in six different breeds. Afrikaner (taurine, South Africa), Kilimanjaro Zebu (indicine, East Africa), Kuri (taurine, West Africa), Shorthorn (taurine, UK), Hariana (indicine, South Asia), and Creole (taurine, South America).


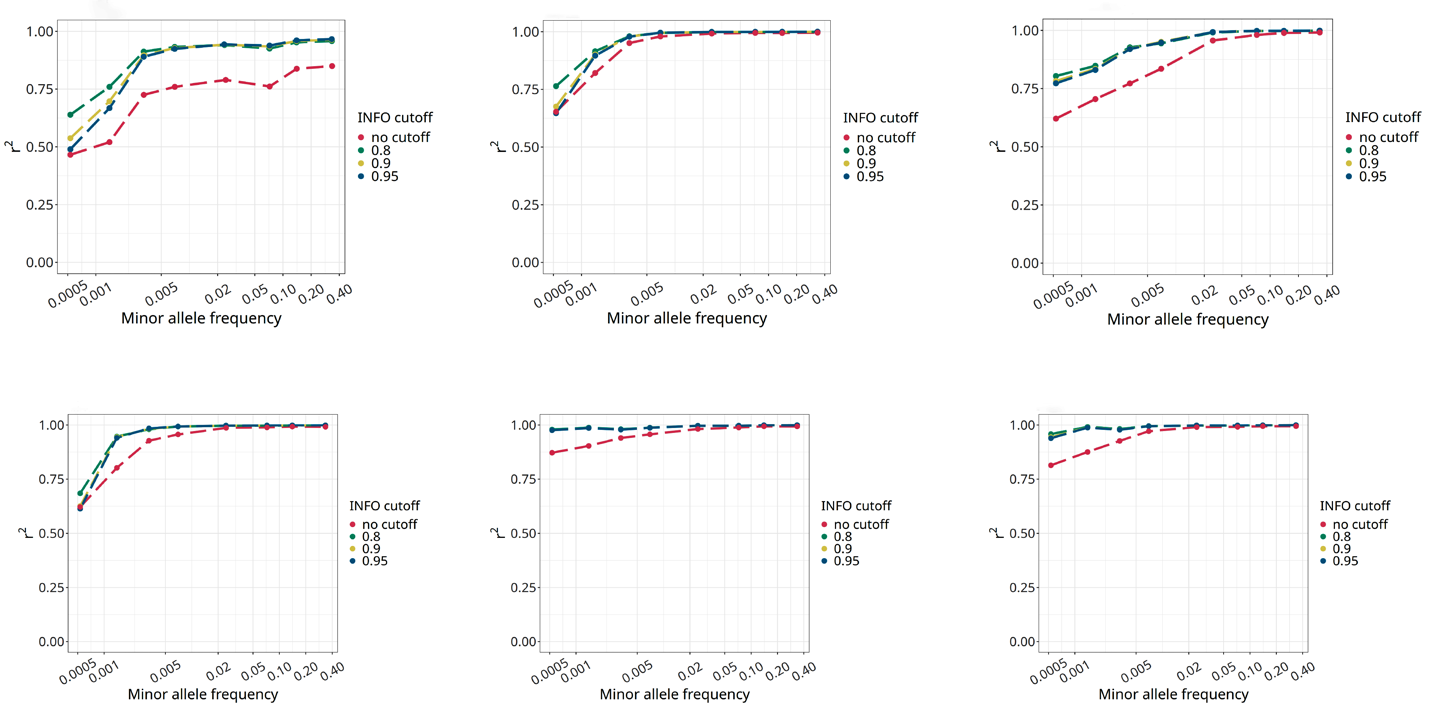


**Supplementary Figure 5**: Evaluation of imputation accuracies and their distributions across MAF, *r*^2^ and INFO scores. Six different cattle breeds are shown that were assessed for imputation accuracy at 4× sequencing depth (Hariana [South Asia], Afrikaner [South Africa], Shorthorn [Europe], Kilimanjaro Zebu [East Africa], Creole [South America], and Kuri [West Africa]).


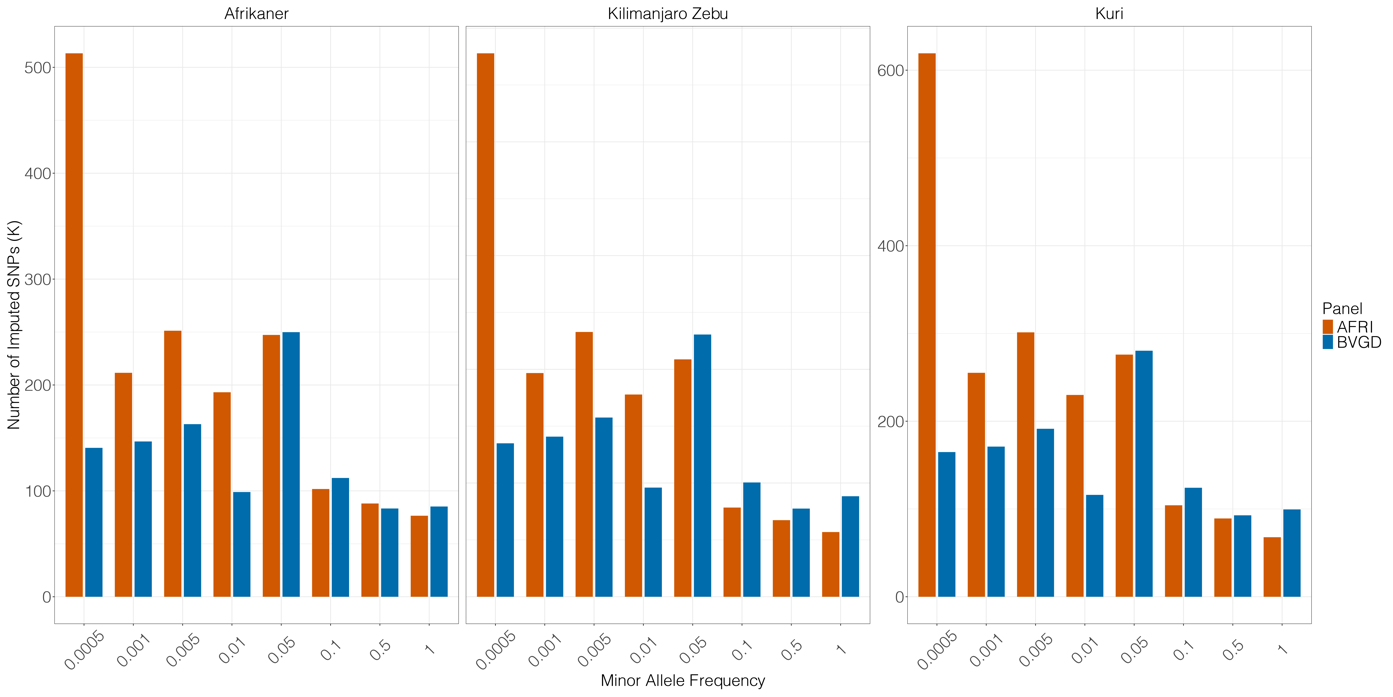


**Supplementary Figure 6**: Evaluation of the number of SNPs distributions across MAF. Three different cattle breeds are shown that were assessed for SNP counts at 0.5× sequencing depth. Afrikaner [South Africa], Kilimanjaro Zebu [East Africa] and Kuri [West Africa].
